# Entropy Generation and Heat Transfer in Drilling Nanoliquids with Clay Nanoparticles

**DOI:** 10.3390/e21121226

**Published:** 2019-12-16

**Authors:** Kottakkaran Sooppy Nisar, Dolat Khan, Arshad Khan, Waqar A Khan, Ilyas Khan, Abdullah Mohammed Aldawsari

**Affiliations:** 1 Department of Mathematics, College of Arts and Sciences, Prince Sattam bin Abdulaziz University, Wadi Al-Dawaser 11991, Saudi Arabia; n.sooppy@psau.edu.sa; 2 Department of Mathematics, City University of Science & Information Technology, Peshawar 25000, Pakistan; dolat.ddk@gmail.com; 3 Institute of Computer Science and Information Technology, The University of Agriculture, Peshawar 25000, Pakistan; arshmaths@gmail.com; 4 Department of Mechanical Engineering, College of Engineering, Prince Mohammad Bin Fahd University, Al Khobar 31952, Saudi Arabia; 5 Faculty of Mathematics and Statistics, Ton Duc Thang University, Ho Chi Minh City 72915, Vietnam; 6 Department of Chemistry, College of Arts and Science-Wadi Al-Dawaser, Prince Sattam bin Abdulaziz University, Alkharj 11991, Saudi Arabia; abdullah.aldawsari@psau.edu.sa

**Keywords:** entropy generation, heat transfer, drilling nanoliquid, clay nanoparticles, Maxwell–Garnett (MG) and Brinkman models, different base fluids and water cleaning

## Abstract

Different types of nanomaterials are used these days. Among them, clay nanoparticles are the one of the most applicable and affordable options. Specifically, clay nanoparticles have numerous applications in the field of medical science for cleaning blood, water, etc. Based on this motivation, this article aimed to study entropy generation in different drilling nanoliquids with clay nanoparticles. Entropy generation and natural convection usually occur during the drilling process of oil and gas from rocks and land, wherein clay nanoparticles may be included in the drilling fluids. In this work, water, engine oil and kerosene oil were taken as base fluids. A comparative analysis was completed for these three types of base fluid, each containing clay nanoparticles. Numerical values of viscosity and effective thermal conductivity were computed for the nanofluids based on the Maxwell–Garnett (MG) and Brinkman models. The closed-form solution of the formulated problem (in terms of partial differential equations with defined initial and boundary conditions) was determined using the Laplace transform technique. Numerical facts for temperature and velocity fields were used to calculate the Bejan number and local entropy generation. These solutions are uncommon in the literature and therefore this work can assist in the exact solutions of a number of problems of technical relevance to this type. Herein, the effect of different parameters on entropy generation and Bejan number minimization and maximization are displayed through graphs.

## 1. Introduction

The use of the second law of thermodynamics to analyze heated fluid flow in engineering devices and systems has become noteworthy. In thermal science, it has been observed that a significant amount of energy is wasted with heat transfer. As a result, many researchers have realized that such energy losses or entropy generation can be minimized by properly designing a system. Entropy generation is produced by many sources, such as heat transfer in a thermal system. In an engineering system, entropy generation is induced by numerous sources. Key sources of entropy generation in thermal systems are viscous dissipation, mass transfer, heat transfer, chemical reaction and electrical conduction, which have been deliberated by Bejan and co-authors in a series of revolutionary publications [1,2,3,4]. Recently, Khan et al. [5] investigated entropy generation for magnetohydrodynamic (MHD) conjugate flow. The exact analysis was obtained through a Laplace transform approach and discussed graphically. Awed [6] investigated a new definition of the Bejan number. The Bejan number is useful because it can provide evidence about the dominance of a magnetic field and fluid friction entropy through heat transfer, or vice versa. An extended form of the Bejan number to a general form was investigated by Awad and Lage in Reference [7]. Saouli and Aïboud-Saouli [8] analyzed entropy generation in a liquid film falling along an inclined plate. Mahmud et al. [9] reported the same analysis for a mixed convection flow, with the additional influence of a magnetic field. Entropy generation for the natural convection flow of a nanofluid was examined by Selimefendigil et al. [10]. They numerically studied entrapped trapezoidal cavities filled with CuO and Al_2_O_3_ nanoparticles and water-based nanofluids. An analysis of entropy generation for the Tiwari and Das model was reported by Sheremet et al. [11], wherein they carried out some computational work to find a solution. Their main finding was that if nanoparticles were inserted into a fluid, heat transfer was enhanced and, consequently, the cavity of convective flow was reduced. For turbulence-forced convection, entropy generation was discussed in a work by Ji et al. [12]. Recently, Qing et al. [13] studied entropy for Casson nanofluids with the influence of MHD, in which the fluid flowed over a porous surface with a stretching or shrinking sheet. A successive linearization method was used to solve a number of equations and highlighted the influence of various parameters on velocity and temperature. Hayat et al. [14] examined entropy generation for two different nanoparticles: copper and silver. Nonlinear stretching characteristics of the rotating disk employed in the study were taken out with the consideration of water. Farshad and Sheikholeslami [15] studied entropy generation for the purpose of enhancing the performance of solar collectors. In their study, nanofluids containing five different types of nanoparticles were considered. They found that aluminum oxide had greater influence on the velocity of a water-based fluid. Recently, Saqib et al. [16] investigated entropy generation for generalized nanofluids in which a fractional calculus approach was employed in both the formulation and solution.

Recently, researchers have been working to use nanofluids for the improvement of thermal equipment and theoretical and experimental heat transfer. At the same time, industries have been involved in using nanofluids for the enhancement of thermal conductivity, employing different procedures to adjust and characterize the thermophysical properties of nanofluids (e.g., viscosity, thermal conductivity, specific heat capacity and density) [17]. Nanofluids are used for heat transportation in industries which feature hyperthermia, power generation, air conditioning, ventilation, microfabrication and transportation [18,19,20,21]. More specifically, they are used in the cooling and heating systems employed in solar energy. A mixed convection flow for nanofluids was investigated by Ahmed and Khan [22], wherein the Maxwell–Garnett and Brinkman models were used to calculate the thermal conductivity and viscosity of the nanofluids. They performed an exact analysis for two different types of nanoparticles. Further, generalized Brinkman-type nanofluids (a fractional model with non-singular kernel) were reported in a work by Ali et al. [23]. Nanoparticles of different shapes were used to influence the performance of kerosene oil and engine oil. The same nanoparticles were used in a water-based fluid for the investigation of convective heat transfer by Hussanan et al. [24]. The application of nanofluids in evaporating and solar energy systems has also been discussed in the literature [25,26]. In recent years, convective heat transfer in a nanofluid used in drilling was studied by Khan et al. [27], wherein clay nanoparticles were used in the cleaning process.

The present study focused on minimizing entropy generation or energy degradation of clay nanoparticles in working fluids, specifically water, engine oil and kerosene oil-based nanofluids. Herein, the effects of the Bejan number on the drilling fluids are discussed. The effects of different embedded parameters toward entropy generation are also highlighted. Importantly, to our knowledge, no study has yet examined entropy generation in clay nanoparticles. As such, this paper will provide a basis for thermal engineers, contributing toward minimization of useful energy losses. In this article, we used clay nanoparticles in the working nanofluids. The idea of clay nanoparticles in fluid is rarely used. Thus, the use of nanoliquids with clay nanoparticles is a novel idea and rich in terms of possible applications. Clay nanoparticles have numerous applications in biological and medical sciences. They are also used in water purification because they can absorb impurities from water, thereby cleaning it. In other sciences, several articles have been published on clay nanoparticles; however, in the literature concerning fluids and particularly from a theoretical perspective, this idea has not been used. To be specific, we mean the exact and numerical sides of the theoretical point of view. Therefore, we suggest this article may be of interest to many researchers.

## 2. Model Formulation

Unsteady flow of water, engine oil and kerosene oil-based nanofluids with $y_{1}>0,$ region were considered. The plate was heated and fixed with x−axis at x=0 and y−axis was normal to the plate. Initially, the plate and nanofluid were stationary with constant temperature $\Theta_{\infty }$. After a certain amount of time, the plate started to move in the direction of its plane $y_{1}=0$ with initial velocity $U_{0}$ and the nanofluid temperature was raised up to $\Theta_{w}$. Under the usual Boussinesq approximation, the equations governing flow were:

(1) $$\rho_{nf}\frac{\partial u_{1}}{\partial t_{1}}=\mu_{nf}\frac{\partial^{2}u_{1}}{\partial y_{1}{}^{2}}+g{\left(\rho \beta_{\Theta }\right)}_{nf}\left(\Theta -\Theta_{\infty }\right).$$

Here $u_{1}=u_{1}(y_{1},t_{1})$ and $\Theta_{1}=\Theta_{1}(y_{1},t_{1})$.

Brinkman [28] suggested the subsequent relation among dynamic viscosity of the base fluid and nanofluid:

(2) $$\mu_{nf}=\frac{\mu_{f}}{{\left(1-\phi \right)}^{2.5}}.$$

Based on the Maxwell–Garnett (MG) model, the following expression for the density of a nanofluid was used (Khan et al. [29], Matin and Pop [30]):

(3) $$\rho_{nf}=\left(1-\phi \right)\rho_{f}+\phi \rho_{s}.$$

The energy equation was:

(4) $${\left(\rho C_{p}\right)}_{nf}\frac{\partial \Theta }{\partial t_{1}}\left(y_{1},t_{1}\right)=K_{nf}\frac{\partial^{2}\Theta }{\partial y_{1}{}^{2}}\left(y_{1},t_{1}\right),$$

where $K_{nf}$ and ${\left(\rho C_{p}\right)}_{nf}$ are the thermal conductivity and heat capacity of the nanofluids, defined as:

(5) $$\frac{K_{nf}}{K_{f}}=\frac{K_{s}+2K_{f}-2\phi \left(K_{f}-K_{s}\right)}{K_{s}+2K_{f}+\phi \left(K_{f}-K_{s}\right)},\;\;\;{\left(\rho C_{p}\right)}_{nf}=\left(1-\phi \right){\left(\rho C_{p}\right)}_{f}+\phi {\left(\rho C_{p}\right)}_{s}\;\;\;.$$

The physical initial and boundary conditions were:

(6) $$u\left(y_{1},0\right)=0,\;\;\Theta \left(y_{1},0\right)=\Theta_{\infty }\;\;\mathrm{for}\;\mathrm{all}\;\;y_{1}\geq 0,\;$$

(7) $$u\left(0,t_{1}\right)=U_{0},\;\;\;\Theta \left(0,t_{1}\right)=\Theta_{w},\;\;t_{1}>0,\;$$

(8) $$u\left(\infty ,t_{1}\right)\to 0,\;\;\;\Theta \left(\infty ,t_{1}\right)\to \Theta_{\infty },\;\;t_{1}>0.$$

For non-dimensionalization, we introduced the subsequent dimensionless variables:

(9) $$y^{*}=\frac{U}{\nu_{f}}y_{1},\;\;t^{*}=\frac{U^{2}}{\nu_{f}}t_{1},\;\;u^{*}=\frac{u}{U},\;\;\;\theta =\frac{\Theta -\Theta_{\infty }}{\Theta_{w}-\Theta_{\infty }},$$

into Equations (1) and (4), giving:

(10) $$\left(\left(1-\phi \right)+\phi \frac{\rho_{s}}{\rho_{f}}\right)\frac{\partial u}{\partial t_{1}}\left(y_{1},t_{1}\right)=\left(\frac{1}{{\left(1-\phi \right)}^{2.5}}\right)\frac{\partial^{2}u}{\partial y_{1}{}^{2}}\left(y_{1},t_{1}\right)+\left(\left(1-\phi \right)+\phi \frac{{\left(\rho \beta_{\Theta }\right)}_{s}}{{\left(\rho \beta_{\Theta }\right)}_{f}}\right)Gr\theta \left(y_{1},t_{1}\right),\;$$

(11) $$\left(\left(1-\phi \right)+\phi \frac{{\left(\rho C_{p}\right)}_{s}}{{\left(\rho C_{p}\right)}_{f}}\right)\frac{\partial \theta \left(y_{1},t_{1}\right)}{\partial t_{1}}=\frac{1}{\mathrm{Pr}}\left(\frac{K_{nf}}{K_{f}}\right)\frac{\partial^{2}\theta \left(y_{1},t_{1}\right)}{\partial y_{1}{}^{2}}.$$

(12) $$u\left(y_{1},0\right)=0,\;\;\;\theta \left(y_{1},0\right)=0\;\;\mathrm{for}\;\mathrm{all}\;y_{1}\geq 0,\;$$

(13) $$u\left(0,t_{1}\right)=1,\;\;\theta \left(0,t_{1}\right)=1,\;\;t_{1}>0,\;$$

(14) $$u\left(\infty ,t_{1}\right)\to 0,\;\;\;\theta \left(\infty ,t_{1}\right)\to 0,\;\;t_{1}>0,$$

where

$$Gr=\frac{g{\left(v\beta_{\Theta }\right)}_{f}{\left(\Theta_{w}-\Theta \right)}_{\infty }}{U^{3}},\mathrm{Pr}=\frac{{\left(\mu C_{p}\right)}_{f}}{K_{f}}.$$

## 3. Entropy Generation (Irreversibility Analysis)

For heat transfer, the dimensionless form of the volumetric rate of entropy generation [31] is given by:

(15) $$Ns=\left(\frac{K_{s}+2K_{f}-2\phi \left(K_{f}-K_{s}\right)}{K_{s}+2K_{f}+\phi \left(K_{f}-K_{s}\right)}\right){\left(\frac{\partial \theta \left(y,t\right)}{\partial y}\right)}^{2}+\frac{Br}{\Omega {\left(1-\phi \right)}^{2.5}}{\left(\frac{\partial u\left(y,t\right)}{\partial y}\right)}^{2}$$

where $B_{r}$ and Ω is the Brinkman number and dimensionless temperature which are defined as:

$$B_{r}=\frac{U^{2}\mu_{f}}{\kappa_{f}\Delta \Theta },\;\;\Omega =\frac{\Delta \Theta }{\Theta_{\infty }},Ns=\frac{Sgen}{E_{0}};E_{0}=\frac{\kappa_{f}U^{2}\Delta^{2}\Theta }{{\Theta_{\infty }}^{2}{v_{f}}^{2}}$$

Equation (15) can be expressed as the sum of entropy generation because of heat transfer ($N_{H}$) and by fluid friction ($N_{F}$). i.e.,

(16) $$Ns=N_{H}+N_{F}.$$

Additionally, the Bejan number, $B_{e}$, is defined as:

(17) $$B_{e}=\frac{N_{H}}{Ns}.$$

The Bejan number gives an idea of the effect of fluid friction and magnetic field control over heat transfer. According to Equation (17), the Bejan number range is between 0 and 1. Whereas $B_{e}>1$ indicates that the irreversibility is only because of fluid friction, both fluid friction and heat transfer have a similar contribution to entropy generation when $B_{e}=1$. When $B_{e}=0.5$, heat transfer and fluid flow irreversibility are of identical significance according to Khan et. al. [32].

## 4. Solution of the Model

By implementing the Laplace transform method, the exact solutions of Equations (10) and (11) under conditions (12) to (14) are [27]:

(18) $$u\left(y_{1},t_{1}\right)=\Psi_{1}\left(y_{1}\sqrt{\frac{a_{1}}{a_{3}}},t_{1}\right)+a_{6}\left[\Psi_{2}\left(y_{1}\sqrt{\frac{a_{1}}{a_{3}}},t_{1}\right)\right]-a_{6}\left[\Psi_{2}\left(y_{1}\sqrt{\frac{a_{4}}{a_{5}}},t_{1}\right)\right],$$

(19) $$\theta \left(y_{1},t_{1}\right)=\Psi_{1}\left(y_{1}\sqrt{\frac{a_{4}}{a_{5}}},t_{1}\right),$$

where

$$\Psi_{1}\left(\zeta ,t_{1}\right)=\mathrm{erfc}\left(\frac{\zeta }{2\sqrt{t_{1}}}\right),\Psi_{2}\left(\zeta ,t_{1}\right)=\left(t_{1}+\frac{\zeta^{2}}{2}\right)\mathrm{erfc}\left(\frac{\zeta }{2\sqrt{t_{1}}}\right)-\zeta \sqrt{\frac{t_{1}}{\pi }}e^{-\;\frac{\zeta^{2}}{4t_{1}}},$$

$$\begin{array}{c} a_{1}=\left(1-\phi \right)+\phi \frac{\rho_{s}}{\rho_{f}},a_{2}=\left(1-\phi \right)+\phi \frac{{\left(\rho \beta_{\Theta }\right)}_{s}}{{\left(\rho \beta_{\Theta }\right)}_{f}},a_{3}=\frac{1}{{\left(1-\phi \right)}^{2.5}},a_{4}=\left(1-\phi \right)+\phi \frac{{\left(\rho C_{p}\right)}_{s}}{{\left(\rho C_{p}\right)}_{f}},\;\\ a_{5}=\frac{1}{\mathrm{Pr}}\left(\frac{K_{nf}}{K_{f}}\right),a_{6}=\frac{a_{2}a_{5}Gr}{a_{3}a_{4}-a_{1}a_{5}},a_{7}=\frac{Gr}{\mathrm{Pr}-1}. \end{array}$$

### 4.1. Solutions for Conventional Base Fluids (Water, Engine Oil and Kerosene Oil)

By taking ϕ=0, Equations (18) and (19) condense to the corresponding solutions for conventional base fluids:

(20) $$u\left(y_{1},t_{1}\right)=\Psi_{1}\left(y_{1},t_{1}\right)+a_{7}\left[\Psi_{2}\left(y_{1},t_{1}\right)\right]-a_{7}\left[\Psi_{2}\left(y_{1}\sqrt{\mathrm{Pr}},t_{1}\right)\right].$$

(21) $$\theta \left(y_{1},t_{1}\right)=\Psi_{1}\left(y_{1}\sqrt{\mathrm{Pr}},t_{1}\right).$$

Note: Equations (20) and (21) collectively represent the fluid velocity and energy transfer for all three types of fluids (water, engine oil, and kerosene oil). However, during computational analysis, the results for each fluid (tabular or graphical) can be obtained separately using their respective thermophysical properties, outlined in Table 1.

## 5. Graphical Results and Physical Interpretations

In this paper, the study of entropy generation in drilling nanofluids with clay nanoparticles was investigated using the Maxwell–Garnett and Brinkman models. Analytical results for temperature and velocity were gained via the Laplace transform technique. Herein, the impact of irreversibility analysis and Bejan number is discussed graphically. The thermophysical properties of clay nanoparticles with water, engine oil and kerosene oil-based fluids are specified in Table 1. The influence of different flow parameters on temperature, velocity, Bejan number and entropy generation are shown graphically and summarized in the subsequent paragraphs.

**Figure 1:**
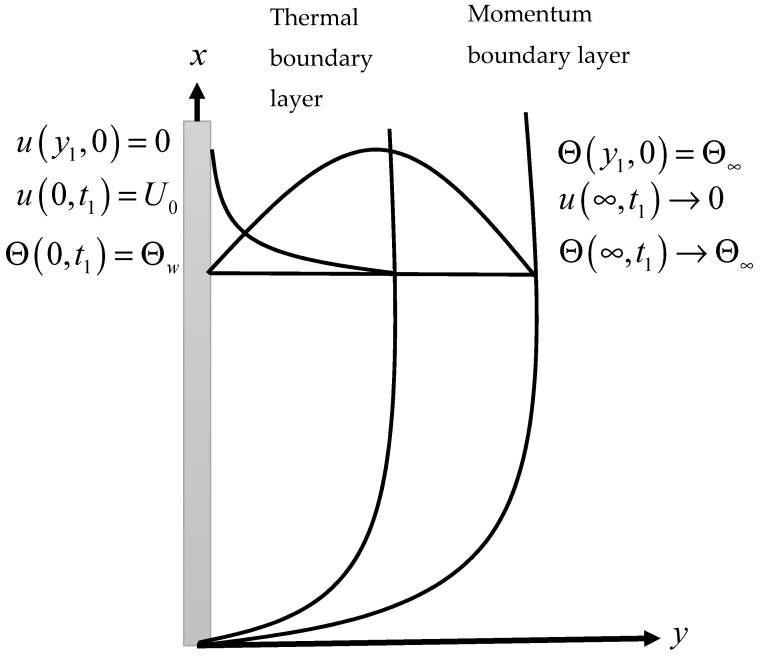
Physical sketch of the problem.

**Figure 2:**
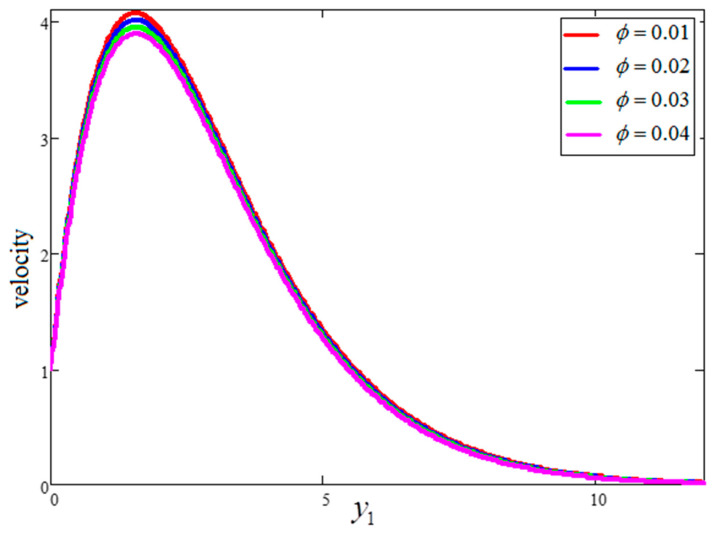
Velocity variation for different values of ϕ, where $\;t_{1}=1,\;\;Gr=10,\;\;\mathrm{Pr}=6.21$.

**Figure 3:**
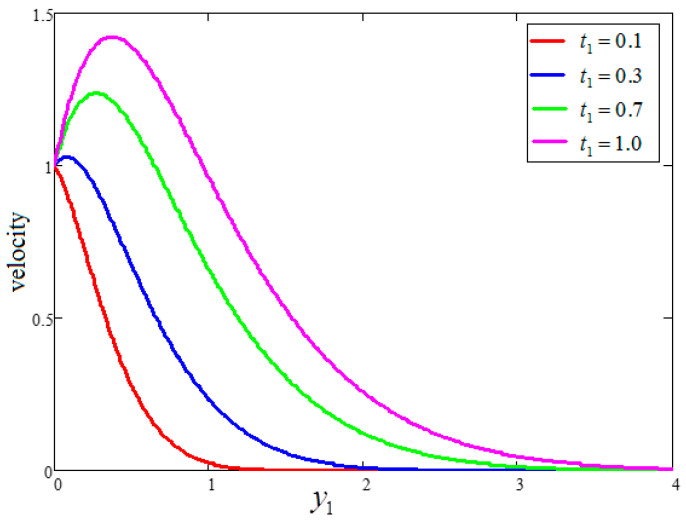
Velocity variation for different values of $t_{1}$, where ϕ=0.04,  Gr=10,  Pr=6.21.

A physical sketch of the problem is given in Figure 1. The influence of clay nanoparticles volume fraction “ϕ” on velocity is deliberated in Figure 2. As seen in the figure, an increase in the volume fraction “ϕ” leads to a decrease in the velocity. It is observed that for pure water, the velocity is at a maximum at “ϕ=0”, while at a minimum for “ϕ=0.04”. The viscous forces rise when the value of “ϕ” becomes greater, resulting in a decrease in the velocity. It is clear that a water-based fluid with clay nanoparticles is denser than pure water. Figure 3 shows the influence of time “$t_{1}$” on the velocity profile. The velocity rises with time because of unsteady fluid. This is physically true as the fluid is initially at rest and, with increasing time, its motion increases for large values of the independent variable, y. However, for very large values of y—that is, when y goes to infinity—the fluid velocity decays to zero. Indeed, this is because of the imposed second boundary condition of velocity.

**Figure 4:**
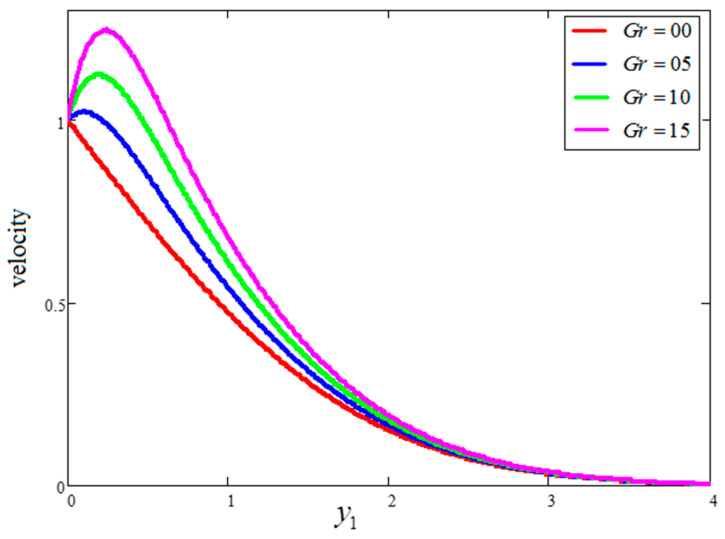
Velocity variation for different values of Gr, where $\phi =0.04,\;\;t_{1}=1,\;\;\mathrm{Pr}=6.21$.

**Figure 5:**
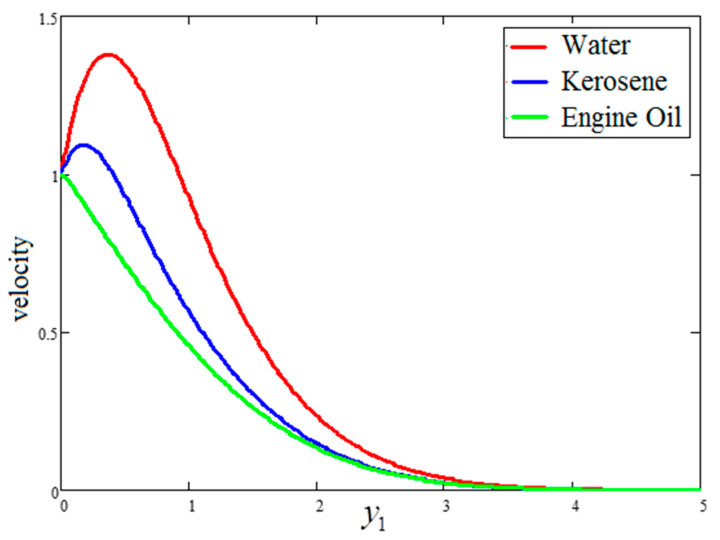
Comparison of velocity variation for different nanofluids, where $\phi =0.04,\;\;t_{1}=1,\;\;Gr=10.$.

**Figure 6:**
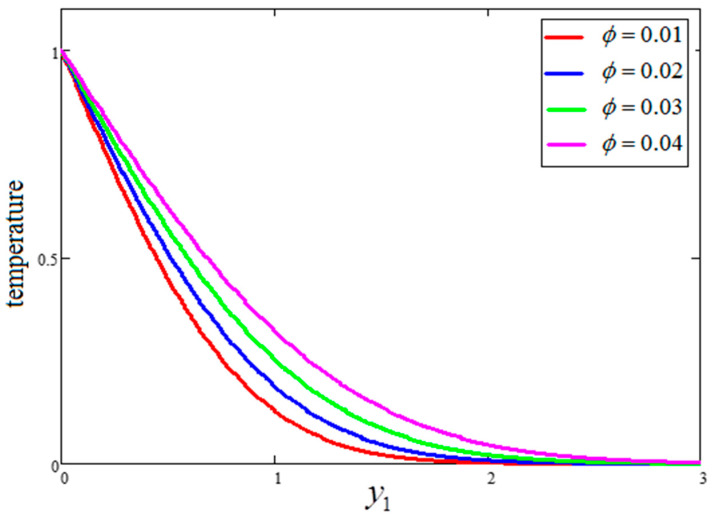
Temperature variation for different ϕ, where $\;t_{1}=1,\;\;\mathrm{Pr}=6.21$.

The effect of “Gr” on velocity is presented in Figure 4, wherein an increase in the velocity profile for increasing values of “Gr” is evident. Actually, the buoyancy force is increased and the viscous force is decreased for greater values of Gr, resulting in an increase in the velocity profile. A comparison of the velocity profiles for water, engine oil and kerosene oil-based fluids with clay nanoparticles is highlighted in Figure 5. As seen in the figure, the velocity profile of the engine oil-based nanofluid is less than that of the kerosene oil and water-based nanofluids. These trends occurred because of the dissimilar thermal conductivities of the base fluids. Comparatively, the engine oil had lower thermal conductivity than the kerosene oil and water-based nanofluids. The temperature variation for four different values of “ϕ” is highlighted in Figure 6. As evidenced in the figure, larger values of “ϕ” leads to enhanced thermal conductivity. Consequently, the thickness of thermal boundary rubbish increases the temperature profile. The results obtained for the effects of ϕ, $t_{1}$ and Gr are quite identical to those published by Khan et al. [27].

**Figure 7:**
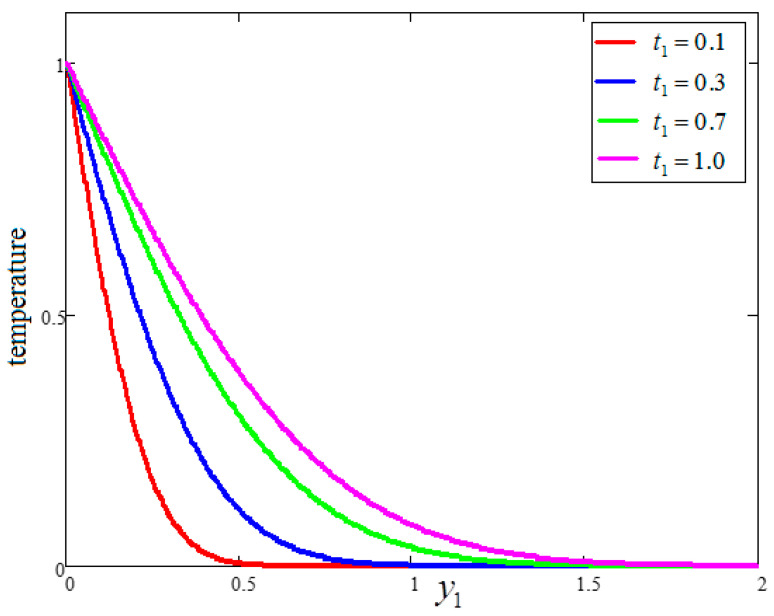
Temperature variation for different for different $t_{1}$, where ϕ=0.04, Pr=6.21.

**Figure 8:**
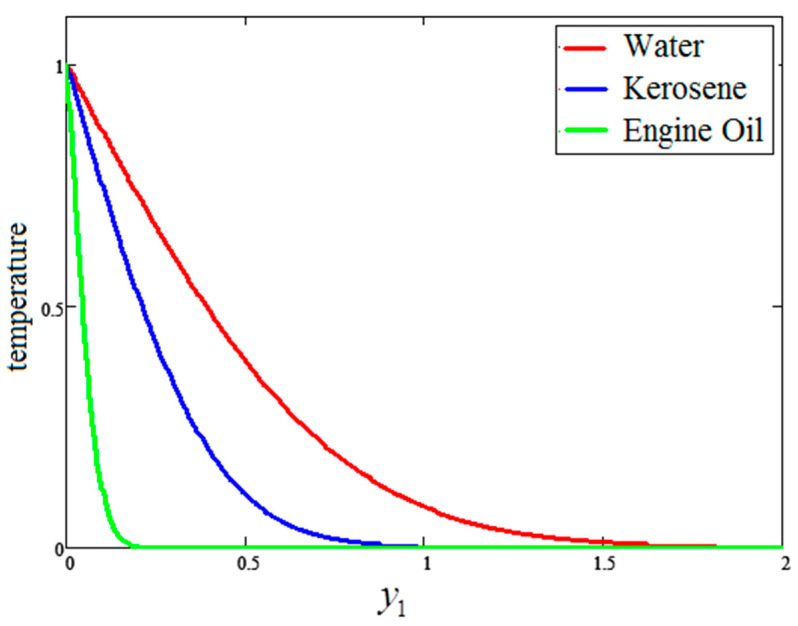
Comparison of temperature variation for different nanofluids, where $\phi =0.04,\;\;t_{1}=1.$

Figure 7 illustrates the impact of temperature for different values of time “$t_{1}$”, wherein the temperature profile increases with the passage of time. Figure 8 provides a comparison of the temperature profiles for the nanofluids with different bases. It is observed that the temperature profile for water is greater than that of kerosene oil and engine oil, which is identical to the results obtained by Khan et al. [27].

**Figure 9:**
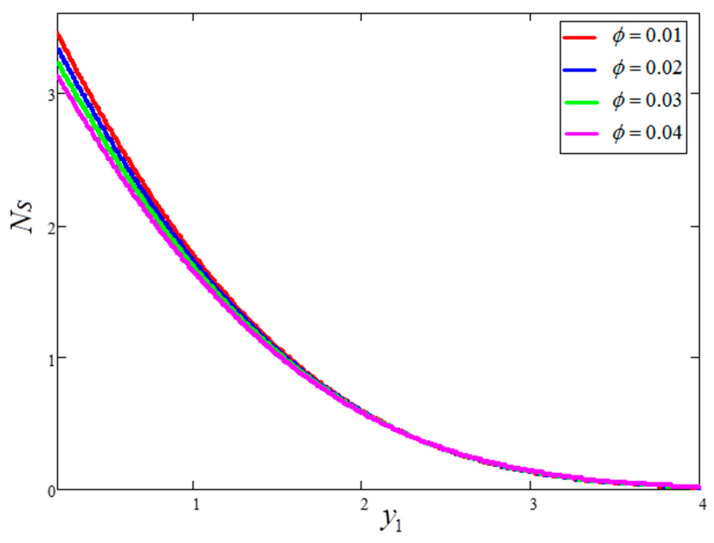
Entropy generation for different values of ϕ, where $\;\;t_{1}=1,\;\;Gr=10,\;\;\mathrm{Pr}=6.21,\;\;\Omega =10,\;\;Br=0.1$.

**Figure 10:**
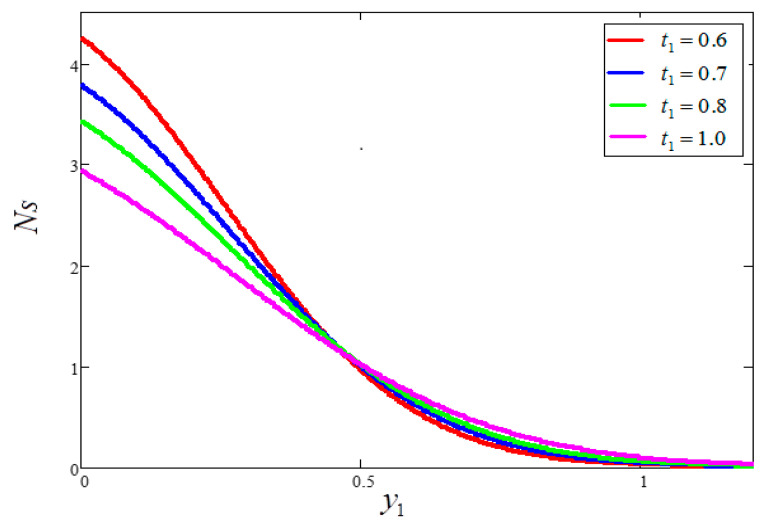
Entropy generation for different values of $t_{1}$, where ϕ=0.04,  Gr=10,  Pr=6.21,  Ω=10,  Br=0.1.

**Figure 11:**
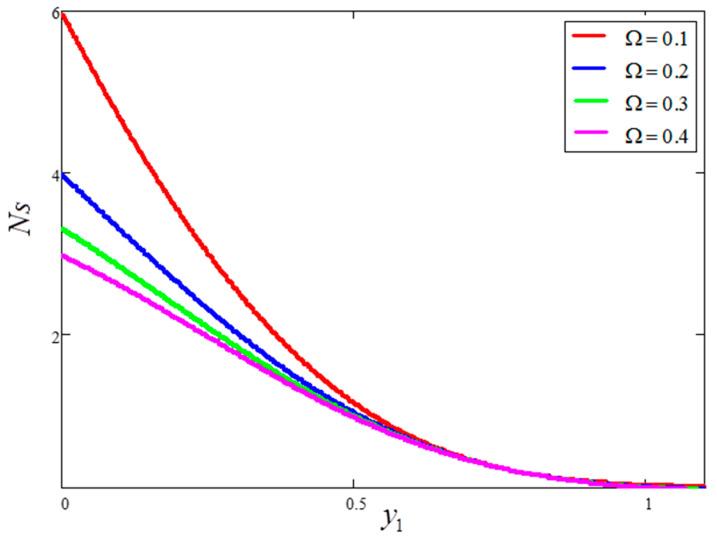
Entropy generation for different values of Ω, where $\phi =0.04,\;\;t_{1}=1,\;\;Gr=10,\;\;\mathrm{Pr}=6.21,\;\;Br=0.1$.

**Figure 12:**
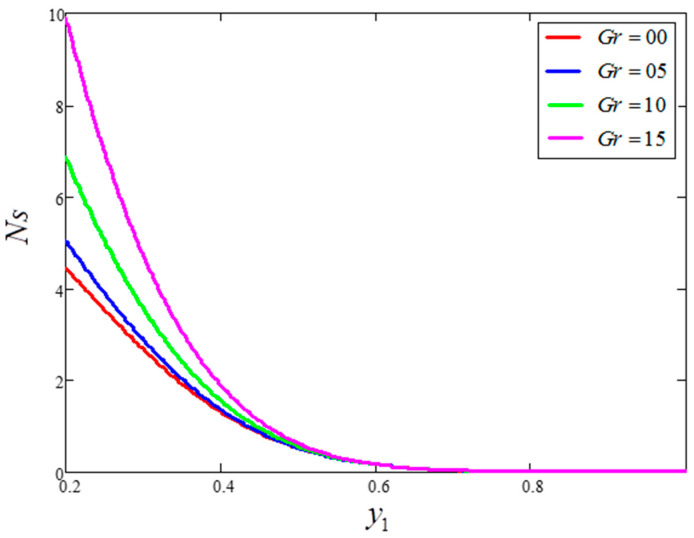
Entropy generation for different values of Gr, where $\phi =0.04,\;\;t_{1}=1,\;\;\mathrm{Pr}=6.21,\;\;\Omega =10,\;\;Br=0.1$.

**Figure 13:**
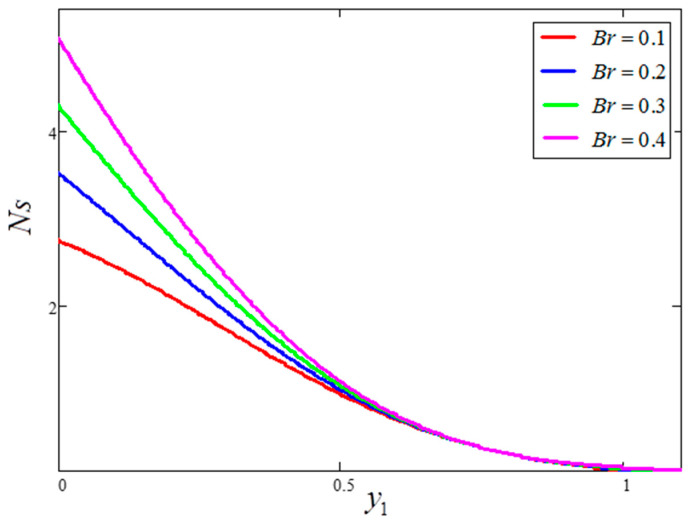
Entropy generation for different values of Br, where $\phi =0.04,\;\;t_{1}=1,\;\;Gr=10,\;\;\mathrm{Pr}=6.21,\;\;\Omega =10$.

**Figure 14:**
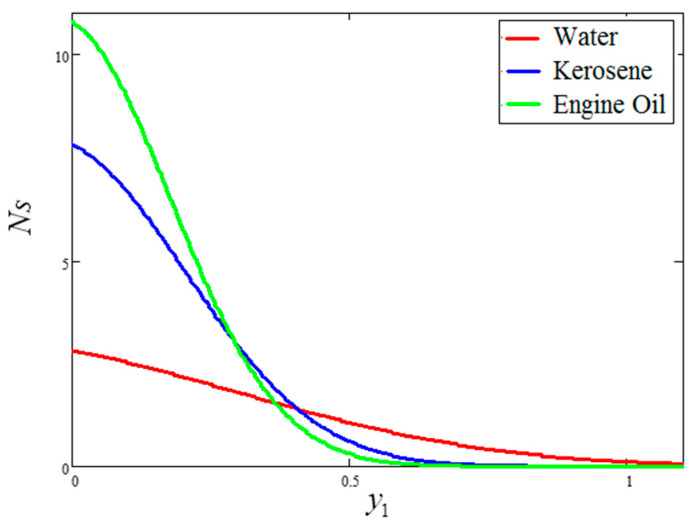
Comparison of entropy generation for different nanofluids, where $\phi =0.04,\;\;t_{1}=1,\;\;Gr=10,\;\;\;\Omega =10,\;\;Br=0.1$.

The impact of entropy generation for dissimilar values of volume fraction “ϕ” clay nanofluid is plotted in Figure 9. For greater values of ϕ, thermal conductivity increases and, as a result, entropy generation decreases. Figure 10 highlights the influence of entropy generation for “$t_{1}$”, in which an increase in “$t_{1}$” leads to a decrease in entropy generation. Figure 11 presents the entropy generation for different values of Ω, wherein Ω is defined as the temperature difference. The figure shows that an increase in temperature difference is associated with a decrease in entropy generation. Figure 12 displays the influence of entropy generation for unlike values of Gr. For greater values of Gr, the buoyancy force increases, resulting in an increase in entropy generation. It is noted that, from this, an increase in Gr could save energy in the system. The influence of Brinkman’s number “Br” is investigated in Figure 13. Brinkman’s number is the ratio of heat produced by viscous dissipation to heat transfer by conduction. According to the figure, a large value of Brinkman’s number produced a high amount of heat via viscous dissipation, and vice versa. Therefore, high values of Brinkman’s number were associated with a rise in entropy generation. Figure 14 provides a comparison of the three working nanofluids used in this work in terms of entropy generation. It is seen that water has smaller entropy generation compared to engine and kerosene oils. This is because water has greater thermal conductivity than the other fluids. The influence of ϕ, Br, $t_{1}$, Gr and Ω on entropy generation is similar to the graphical results obtained by Khan et al. [33] and Saqib et al. [16]. 

**Figure 15:**
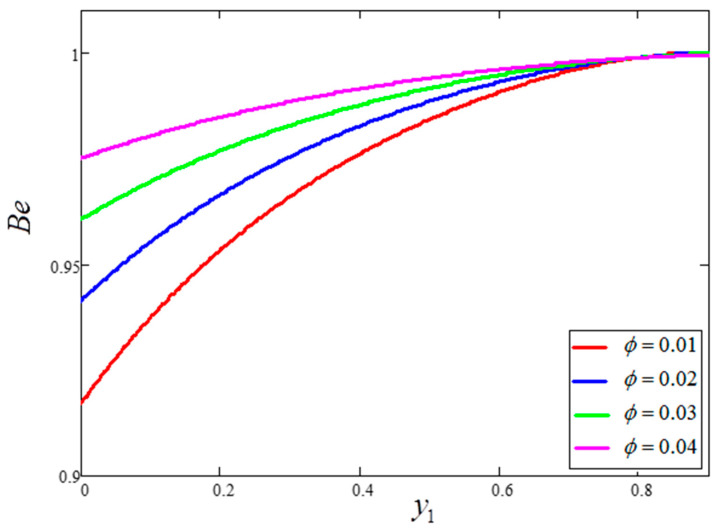
Bejan number variation for different values of ϕ,where $\;t_{1}=1,\;\;Gr=10,\;\;\mathrm{Pr}=6.21,\;\;\Omega =10,\;\;Br=0.1$.

**Figure 16:**
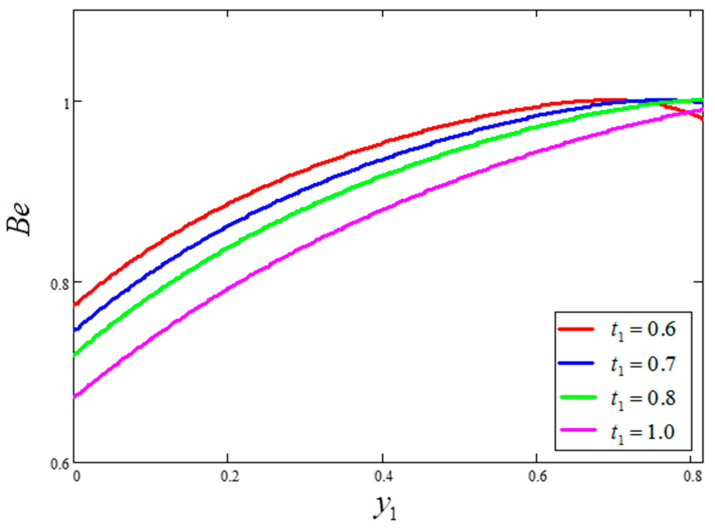
Bejan number variation for different values of $t_{1}$, where ϕ=0.04,  Gr=10,  Pr=6.21,  Ω=10,  Br=0.1.

**Figure 17:**
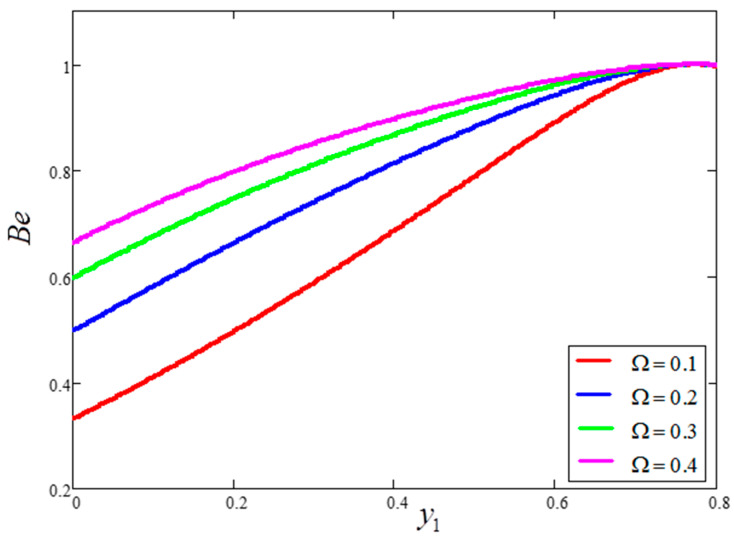
Bejan number variation for different values of Ω, where $\phi =0.04,\;\;t_{1}=1,\;\;Gr=10,\;\;\mathrm{Pr}=6.21,\;\;Br=0.1$.

**Figure 18:**
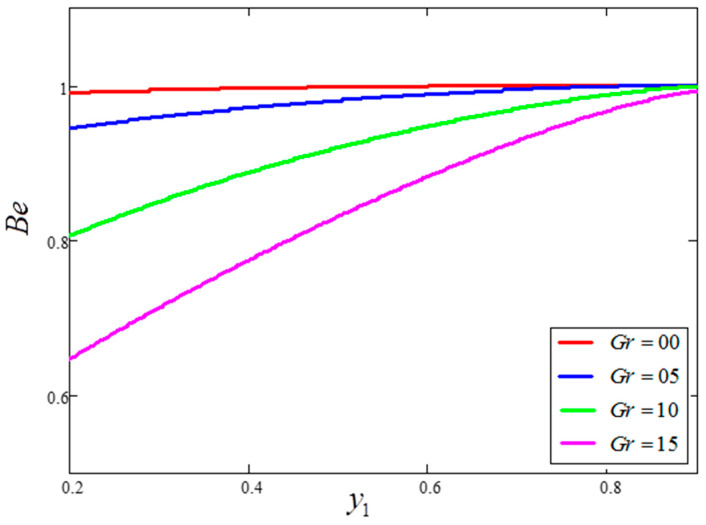
Bejan number variation for different values of Gr, where $\phi =0.04,\;\;t_{1}=1,\;\;\mathrm{Pr}=6.21,\;\;\Omega =10,\;\;Br=0.1$.

**Figure 19:**
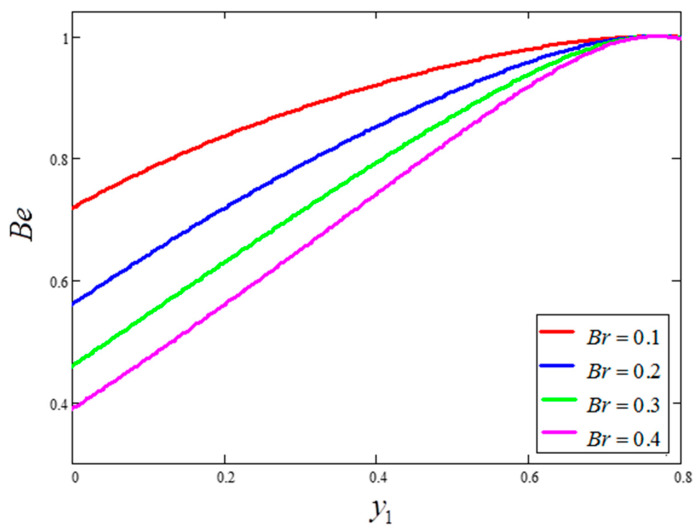
Bejan number variation for different values of Br, where $\phi =0.04,\;\;t_{1}=1,\;\;Gr=10,\;\;\mathrm{Pr}=6.21,\;\;\Omega =10$.

**Figure 20:**
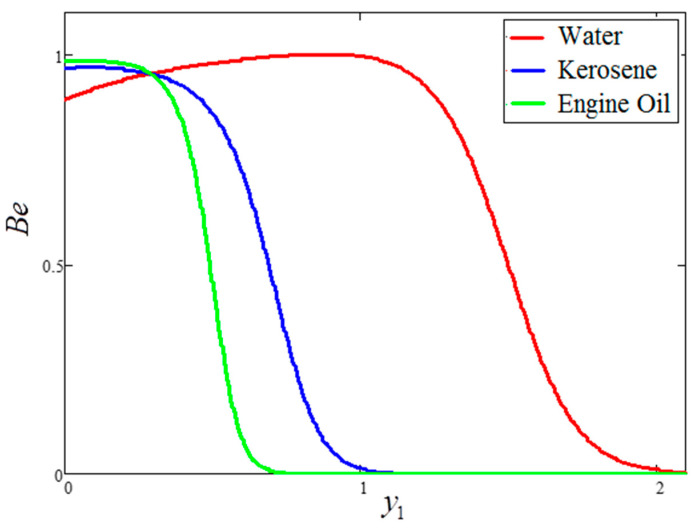
Comparison of Bejan number variation for different nanofluids, where $\phi =0.04,\;\;t_{1}=1,\;\;Gr=10,\;\;\Omega =10,\;\;Br=0.1$.

The influence of volume fraction “ϕ” on the Bejan number of the nanofluids is represented in Figure 15. Evidently, an increase in the volume fraction “ϕ” of nanoparticles leads to a decrease in the influence of the Bejan number. The influence of $t_{1}$ and Ω on Bejan number variation is highlighted in Figure 16 and Figure 17, respectively. From Figure 16, the Bejan number appears to increase with increasing $t_{1}$. However, Ω is found to have the opposite effect, with an increase in Ω leading to an decrease in the Bejan number, as shown in Figure 17. Figure 18 highlights the difference in the Bejan number with respect to changes in Gr, wherein greater values of Gr are correlated with decreased Bejan numbers. This is because heat transfer reunification becomes dominant in the region near to the plate with an increasing value of Gr. Bejan number variation for different values of “Br” is reported in Figure 19. For increasing values of Br, the Bejan number decreases. A comparison of the Bejan numbers associated with the different working nanofluids with clay nanoparticles is shown in Figure 20. Water was found to have the greatest influence on Bejan number. The graphical observations of ϕ, Br, $t_{1}$, Gr and Ω with Bejan number are in agreement with the results obtained by Khan et al. [33] and Saqib et al. [16].

## 6. Conclusions

The entropy generation of different drilling nanofluids with clay nanoparticles is reported. For the nanofluid model, the Tiwari and Das model was considered. Exact solutions for velocity and temperature were evaluated by means of the Laplace transform technique. The most important findings can be summarized as follows:

1. For the water-based clay nanofluid, the velocity, temperature and Bejan number were higher compared to those obtained for the kerosene oil and engine oil-based nanofluids, but lower in the case of entropy generation.
2. The behavior of temperature and Bejan number decreased for greater clay nanoparticle volume fractions “ϕ”. However, velocity and entropy generation showed the opposite behavior.
3. An increase in time “$t_{1}$” increased the velocity and temperature value, as well as reduced the Bejan number and entropy generation value.
4. Gr is a major source of enhancement to the velocity and entropy generation value, although it decreases the Bejan number.
5. Entropy generation is smaller for greater values of Ω and larger for greater values of Br.
6. Bejan number is smaller for greater values of Br and larger for greater values of Ω.

## Figures and Tables

**Table 1:** Thermophysical properties of clay nanoparticles with different base fluids by Khan et al. [27].

Material	Base Fluids			Nanoparticles
	Engine Oil	Kerosene Oil	Water	Clay
$\rho \left(\mathrm{kg}/m^{3}\right)$	884	783	997	6320
$c_{p}\left(J/\mathrm{kg}\;K\right)$	1910	2090	4179	531.8
K(W/m K)	0.114	0.145	0.613	76.5
$\beta \times 10^{-5}\left(K^{-1}\right)$	70	99	21	1.80
Pr	500	21	6.2	-

## Abbreviations

**Nomenclature**		**Greek symbols**			
${\left(C_{p}\right)}_{nf}$	Nanofluid heat capacity at a constant pressure	$kg^{-1}\;\;K^{-1}$	$\beta_{\Theta }$	Volumetric coefficient of thermal expansion	$K^{-1}$
erfc	Complementary error function.		θ	Dimensionless temperature	
Gr	Thermal Grashof number		Θ	Temperature of the fluid	K
g	Acceleration due to gravity	$m\;s^{-2}$	$\Theta_{\infty }$	Ambient temperature	K
$K_{f}$	Base fluid thermal conductivity	$W\;m^{-1}\;K^{-1}$	$\Theta_{w}$	Wall temperature	K
$K_{nf}$	Nanofluid thermal conductivity	$W\;m^{-1}\;K^{-1}$	$\rho_{f}$	Base fluid density	$kg\;\;m^{-3}$
$K_{s}$	Solid particle thermal conductivity	$W\;m^{-1}\;K^{-1}$	$\rho_{nf}$	Nanofluid density	$kg\;\;m^{-3}$
Pr	Prandtl number		$\rho_{s}$	Solid particle density	$kg\;\;m^{-3}$
$t_{1}$	Time	s	ϕ	Nanoparticle volume fraction	
u	Velocity of the fluid	$m\;s^{-1}$	$\mu_{f}$	Base fluid dynamic viscosity	$kg\;\;m^{-1}\;s^{-1}\;$
			$\mu_{nf}$	Nanofluid dynamic viscosity	$kg\;\;m^{-1}\;s^{-1}\;$
